# PI3K Signaling in Neurons: A Central Node for the Control of Multiple Functions

**DOI:** 10.3390/ijms19123725

**Published:** 2018-11-23

**Authors:** Karina Sánchez-Alegría, Manuel Flores-León, Evangelina Avila-Muñoz, Nelly Rodríguez-Corona, Clorinda Arias

**Affiliations:** Departamento de Medicina Genómica y Toxicología Ambiental, Instituto de Investigaciones Biomédicas, Universidad Nacional Autónoma de México, AP 70-228, 04510 México, DF, Mexico; k.aisa.62@hotmail.com (K.S.-A.); manuel.leoncio75@gmail.com (M.F.-L.); avmueva@gmail.com (E.A.-M.); rodriguezcorona.nelly@gmail.com (N.R.-C.)

**Keywords:** PI3K signaling, neurodegeneration, neuronal metabolism, gene expression, neuroinflammation

## Abstract

Phosphoinositide 3-kinase (PI3K) signaling contributes to a variety of processes, mediating many aspects of cellular function, including nutrient uptake, anabolic reactions, cell growth, proliferation, and survival. Less is known regarding its critical role in neuronal physiology, neuronal metabolism, tissue homeostasis, and the control of gene expression in the central nervous system in healthy and diseased states. The aim of the present work is to review cumulative evidence regarding the participation of PI3K pathways in neuronal function, focusing on their role in neuronal metabolism and transcriptional regulation of genes involved in neuronal maintenance and plasticity or on the expression of pathological hallmarks associated with neurodegeneration.

## 1. Introduction

Phosphoinositide 3-kinases (PI3Ks) are a family of multifaceted enzymes that play a central role in diverse metabolic processes that regulate many aspects of cell physiology. PI3Ks are evolutionarily conserved enzymes that transduce mitogenic and metabolic signals to promote cell growth, proliferation, migration, and apoptosis [1,2,3]. PI3Ks belong to a family of lipid kinases that comprise eight isoforms that have been classified according to their substrate specificity and structural features into three classes: I, II, and III [1,2,3,4]. The most studied family is class I, which catalyzes the conversion of phosphatidylinositol into 3′-phosphoinositides (PtdIns-3P), while class II PI3Ks, which consist of a single catalytic subunit, prefer PtdIns-4P and PtdIns-3,4P as substrates, similar to class III PI3Ks, which are additionally involved in vesicular trafficking, secretion, and autophagy [5,6,7].

Class I PI3Ks form a functional heterodimer composed of two subunits, one catalytic and one regulatory, with molecular weights of 110 kDa (p110) and 85 kDa (p85), respectively. The catalytic p110 subunit also has four different isoforms (α, β, γ, and δ, also known as PI3KCA, PI3KCB, PI3KCG, and PI3KCD, respectively), and the most frequent regulatory p85 subunit encoded by three different genes (α, β, and γ) [8]. Most neurons in the hippocampus, cerebral cortex, and cerebellum express both the catalytic p110α and all the regulatory p85 subunits at high levels [9,10]. PI3K activates protein kinase B (PKB), also known as AKT, which in turn activates mTOR. Particularly in the brain regions aforementioned, activation of the AKT/mTOR pathway seems to be essential for neuronal development and synapse formation [11,12,13,14] and contributes to neuronal plasticity and memory performance [15,16,17,18]. The activation of this pathway through receptor tyrosine kinase (RTK) by growth factors constitute the core path that regulates neuronal proliferation, maturation, and integration into mature circuits in the brain [19]. The important function of PI3K in neurons has also been demonstrated for its involvement in severe brain pathologies, such as developmentally-associated brain malformations [20,21], epilepsy [22,23], aging-associated neurodegeneration [24,25,26], and brain cancer [27,28].

Different combinations of PI3K isoforms may result in different functional events depending on the metabolic context, upstream signals, and interconnection of different cellular pathways. For example, in sensory neurons, p110δ is an important signaling component for efficient axonal elongation in the developing and regenerating nervous system [29]. In this sense, the inactivation of the p110δ produces deficient axonal elongation and prevents axonal regeneration in a model of sciatic nerve crush injury [29]. The manipulation of PI3K/AKT signaling has been proven to induce dramatic changes in myelination. In a conditional knockout for PTEN, the phosphatase that reduces the activation of PI3K/AKT, a hypermyelination phenotype is associated with an increase in PtdIns-3P and AKT activation [30]. It has also been confirmed that the increased synthesis of the p110β subunit of PI3K contributes to the anatomical, behavioral, and molecular defects associated with fragile X syndrome [31]. Accordingly, many studies have demonstrated that the activation of PI3K induces cell proliferation, and overexpression of the p85 regulatory subunit inhibits PI3K/AKT signals, impairs neuronal stem cell proliferation, and disturbs neuronal differentiation during the development of mouse cerebral cortex [32]. PI3K dysregulation also plays a significant role in glioblastoma pathogenesis. Pharmacological inhibition of the catalytic subunits p110β and p110α but not p110δ has impaired glioblastoma growth and caused tumor regression in in vivo models [33]. On the other hand, increased PI3K/AKT/mTOR signaling was identified in several human brain samples, strongly implicating this pathway in diverse cases of hemimegaloencephaly, a developmental disorder characterized by brain overgrowth dependent on the increased number of neurons and glial cells [34].

All of these studies indicate that PI3K/AKT is a central node that integrates developmental signals that are necessary for brain development, maintenance, repair, and plasticity during adult life.

## 2. PI3K Signaling in Neuronal Metabolism

PI3K signaling associated with metabolism has been mainly in the hypothalamus, a region that regulates energy balance and glucose homeostasis. Clear implications point to the role of class I PI3K in energy balance in hypothalamic neurons. PI3K/AKT activation by insulin inhibits glucose production in the liver through the activation of ATP-dependent K^+^ channels in the hypothalamus [35,36,37,38]. In fact, the infusion of insulin and the consequent activation of the PI3K pathway is able to suppress glucose production independently of circulating insulin. Recently, it has been shown that different PI3K catalytic subunits are involved in differential responses of hypothalamic neurons by insulin or leptin. For example, while neuronal hyperpolarization induced by insulin and leptin depends on both p110α and p110β PI3K catalytic subunits, the depolarization effect of leptin depends solely upon the PI3K 110β subunit [39]. These differences affect the regulation of energy balance and glucose homeostasis. PI3K/AKT pathway also contributes to glucose uptake and energy metabolism through the regulation of GLUT3 transporter expression, as well as the expression of the glycolytic enzyme phosphofructokinase-1 (PFK-1) during differentiation of cortical neurons [40]. It has been shown that neuronal glucose uptake is not dependent upon insulin/PI3K/AKT signaling; however, after high neuronal activity, the insulin/PI3K/AKT pathway induces GLUT4 translocation to the membrane to increase glucose transport in hippocampal neurons and improve cognitive functioning [41]. Accordingly, insulin administration improves memory task performance. This positive effect on cognition is completely mediated by PI3K activation and the increase in local glucose metabolism [42]. Thus, the insulin/PI3K/AKT/mTOR pathway importantly modulates neuronal plasticity underlying high cognitive functions. In fact, it was recently reported that AKT3 knockout mice exhibit microcephaly, cognitive defects, and reduced protein synthesis in response to long-term potentiation (LTP) via inactivation of mTOR and reduced protein synthesis necessary to sustain plasticity changes [43]. Different isoforms of PI3K have been implicated in synaptic plasticity and cognitive functions. Genetic deletion or overexpression of PI3Kγ disrupts long-term depression (LTD) and reduces spatial learning tasks, while contextual fear memory is not affected [44,45]. Similarly, activation of PI3K in the amygdala was found to be associated with fear conditioning [46].

**Figure 1:**
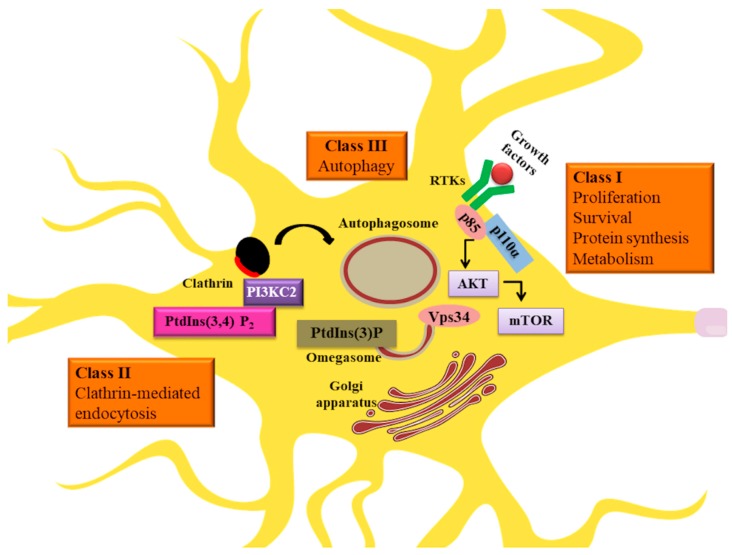
Involvement of different phosphoinositide 3-kinase (PI3K) classes in neuronal function. Class I acting through receptor tyrosine kinase (RTK) pathways mediate survival, proliferation, protein synthesis, and metabolism. Class II participates in vesicular trafficking and class III in autophagy.

Dysregulated PI3K/AKT signaling in neurons has several harmful consequences, such as elevated ROS levels, membrane depolarization, mitochondrial fragmentation, and decreased oxidative phosphorylation and ATP production [47,48,49,50]. It is particularly interesting that the amyloid peptide involved in Alzheimer’s disease (AD) is capable of producing sustained activation of AKT, which in turn phosphorylates the mitochondrial fission protein Drp1. This mechanism has been proposed to be involved in the mitochondrial fragmentation observed in this neurodegenerative disease [47,48,49,50] (Figure 1).

## 3. The Role of PI3K in Neuroinflammation

Inflammation is recognized as a central player in a variety of brain diseases. An exacerbated inflammatory response has been associated with some chronic neuropathological conditions, such as AD [51,52,53,54,55,56]. There is evidence at the systemic level that PI3K induces the activation of NF-κB and, specifically, class I PI3Ks are involved in the transduction pathway of toll-like receptors (TLRs) in immune cells, such as macrophages and dendritic cells. However, in regard to the role of activation of different isoforms of the class I PI3Ks, the results have suggested that they can play either positive or negative roles in the production of pro-inflammatory cytokines [57]. It has been shown that the activation of TLRs can induce the recruitment of class Iα PI3Ks, which leads to downregulation in NF-κB-induced pro-inflammatory cytokines in macrophages [57]. On the other hand, loss of functional PI3Kδ reduces TLR4 internalization and relocation to endosomes, promoting the early secretion of pro-inflammatory cytokines (IL-6 and IL-12) and reducing the secretion of anti-inflammatory cytokines such as interleukin-10 (IL-10) and interferon-β (IFN-β) in dendritic cells [58]. Additionally, it has been shown in vitro and in vivo that PI3Kγ plays an important role in the initial signal transduction events downstream of chemoattractant and chemokine G protein-coupled receptors (receptors for fMLP, C5a, IL-8, LTB4) that promote the extravasation and migration of innate immune cells, such as neutrophils, monocytes, or eosinophil, during inflammation [51,59,60,61,62,63,64,65,66].

In the central nervous system (CNS), microglial cells are resident macrophages involved in immunological responses. Excessive microglia activation may lead to synaptic loss and neuronal dysfunction. It has been shown that the activation of AKT precedes NF-κB-dependent transcription of proinflammatory genes in activated microglia. In this report, the authors found that LPS is able to activate PI3K/AKT through the stimulation of TLR4 [67]. Moreover, using different natural compounds, it has been suggested that PI3Ks are involved in the development of the neuroinflammatory response modulating the release of cytokines. For example, studies in LPS-stimulated microglial cells demonstrated that curcumin attenuates the expression of TNF-α, IL-6, and IL-1β through the suppression of PI3K/AKT-mediated activities [68,69]. Additionally, the flavonoid compound morin exerts anti-inflammatory effects by downregulating MAPK and PI3K/AKT signaling pathways and upregulating the anti-inflammatory PKA/CREB and Nrf2/HO-1 pathways in microglia [70]. Furthermore, it has been shown that TGF-1β, an anti-inflammatory molecule, protects brain tissue by repressing the overactivation of microglial cells via inhibition of PI3K and its downstream signaling molecules [71]. Remarkably, not only are glial cells involved in neuroinflammation, but hippocampal neurons can also contribute to neuroinflammation by releasing TNF-α and IL-1β via TLR4-mediated PI3K/AKT/NF-κB signaling [72].

Chronic inflammation in the brain is a feature of some neurological disorders associated with neuronal damage. Therefore, pharmacological-based therapies focusing on PI3K signaling is a promising research avenue. For example, in an injury model of cerebral ischemia/reperfusion, the use of a class I PI3K inhibitor (ZSTK474) was able to alleviate neurological deficits and reduce the infarct volume. This therapeutic benefit was associated with the induction of an anti-inflammatory phenotype in microglia leading to a decrease in secreted pro-inflammatory molecules through the PI3K/AKT/mTORC1 pathway [73].

In another model of brain injury due to a surgical procedure (SBI), it has been demonstrated that PI3Kγ promotes a pro-inflammatory phenotype. Furthermore, pharmacological inhibitors of PI3Kγ (AS252424 or AS605240) improve neurological function after SBI [74]. Interestingly, chronic neuroinflammation has been associated with the development of AD [53,54,55,56] and a variety of inflammatory intermediaries, including IL-1β, IL-6, and TNF-α, have been found to be upregulated in this disease [51,52,54,55]. Epidemiological studies indicate that anti-inflammatory agents, such as non-steroidal anti-inflammatory drugs (NSAIDs), have a beneficial effect on AD, and several reports have shown that NSAIDs protect against inflammation in transgenic AD models [56]. It has been shown that some NSAIDs can exert their actions by modulating PI3K [75,76,77]. Thus, an interesting line of investigation is now directed at analyzing the relationship between inflammation, PI3K signaling, and AD.

## 4. Genetic and Epigenetic Regulation through the PI3K/AKT Signaling Pathway

One of the main processes that control the genetic programming and functions of neurons is the recruitment and activation of transcription factors and chromatin remodeling complexes in response to specific intrinsic demands and upstream signals. PI3K, through AKT activation, enhances the interaction and activity of several transcription complexes composed of basic helix-loop-helix (bHLH) transcription factors (Neurogenin, NeuroD1, and MASH1), acetyltransferases (HATs), such as CBP and p300, and histone deacetylases (HDACs) [78].

In vitro experiments demonstrate that AKT activation by the natural flavonoid curcumin in combination with an inhibitor of HDACs improves neuronal survival and restores neuronal damage induced by amyloid beta (Aβ), which is a peptide associated with AD [79]. One of the proposed mechanisms for such protection is the AKT-dependent phosphorylation of the transcription factor CREB, which in turn promotes the expression of the brain-derived neurotrophic factor gene *bdnf*. Neuronal survival also seems to be dependent on PI3K/AKT signaling through regulation of the NF-κB transcription factor at two levels: through enhancing the activity of the inhibitory kinase IKK and by directly phosphorylating NF-κB, which increases its activity [80,81].

Experiments in PC12 cells strongly suggest that PI3K/AKT is the signaling pathway responsible for the activation of late phase gene expression involved in neuronal differentiation, neurogenesis, and neuroprotection [82]. Interestingly, nerve growth factor (NGF)-dependent PI3K/AKT/NF-κB signaling is also associated with an epigenetic modification at lysine 9 of histone 3 (H3K9) during neuronal differentiation through the expression of the G protein-coupled delta opioid receptor gene *dor* [83].

Forkhead transcription factors (FOXO) are a family of proteins that play important roles in regulating the expression of genes involved in cell growth, apoptosis, and longevity. When AKT phosphorylates FOXO, it is translocated from the nucleus to the cytosol as an inactive complex, inhibiting transcription of the tumor suppressor p53 and preventing apoptosis, as previously demonstrated in hippocampal neurons after hypoxia [84]. Interestingly, PI3K/AKT signaling is inhibited in neuronal cultures exposed to the Aβ peptide, which induces the translocation of FOXO from the cytosol to the nucleus and the expression of the proapoptotic protein BIM [85]. Recent results have suggested that the neuroprotective effect of the mood stabilizer valproic acid is mediated through the inhibition of glycogen synthase kinase-3 (GSK3) and the activation of PI3K/AKT signaling. Valproic acid is one of the best known epi-drugs and acts as a pan-inhibitor of HDACs that are associated with chromatin remodeling and transcriptional activation of neuroprotective genes, such as fibroblast growth factor (*FGF-21*) mRNA [86].

The balance between acetylation and deacetylation is crucial for the regulation of gene expression in physiological and pathological conditions. Both epigenetic processes are altered during aging and in some neuropathological conditions, such as AD. Studies in primary neuronal cultures and in vivo mouse models of AD at different stages have revealed increased global levels of acetylated histones H3 and H4 [87]. The hyperacetylated state of H3 and H4 was also observed in post-mortem brains of AD patients, especially in the most affected regions, such as the inferior and middle temporal gyrus [88]. Since acetylated histones are associated with open states of the chromatin that allow gene transcription, several genes that play an important role in the progression of AD markers can be upregulated when chromatin is hyperacetylated. One of these genes is the *Bace1* gene for the β-site amyloid precursor protein-cleaving enzyme 1 (BACE1). Upregulation of BACE1 mRNA associated with hyperacetylation in the promoter region of *Bace1* has been reported in the 3xTg-AD mouse model [89,90]. Furthermore, in vitro experiments have demonstrated that this hyperacetylation is associated with a hyperactivated state of the acetyltransferase p300 that not only modifies histones but can recruit transcription factors such as CREB and NF-κB [90].

In addition to acetylation, histones can be mono-, di-, or trimethylated on lysine or arginine residues. However, less is known about how PI3K/AKT can regulate these posttranslational modifications. It has been shown that the polycomb repressive complex 2 is responsible for establishing this epigenetic mark through the methyltransferase enhancer Zeste 2 (EZH2). EZH2 can be regulated by AKT phosphorylation, which reduces its activity and, therefore, the amount of H3K27me3 in the genome [91]. Interestingly, the reduction of this epigenetic marker is associated with the activation of several genes involved in memory, cognition, cell survival, and axonal growth [91,92].

Because epigenetic mechanisms are dynamic and constantly changing, it is not surprising that PI3K/AKT signaling activation in response to growth factors can modulate some of the eraser proteins, such as histone demethylases (KDMs). The phosphorylation induced by AKT promotes the exit of KDM5A from the nucleus and the increase in H3K4me3 content associated with active transcription in specific promoters [91]. Interestingly, it has been shown that brains affected by AD aberrantly accumulate H3K4me3 in the neuronal cytoplasm. This accumulation is associated with the progression of AD-related pathological hallmarks, making a positive correlation as the pathology worsens [93]. These studies suggest that the role of PI3K/AKT participation in AD modifies the transcription of genes related to neuronal function and survival through changes in epigenetic markers.

DNA methyltransferases (DNMTs) are enzymes that are responsible for the addition of a methyl group to the C5 cytosine residue to produce 5-methylcytosine (5mC). During the development of neural stem cells (NSCs), inhibition of the PI3K/AKT signaling pathway enhances the expression of DNMT1 and DNMT3a and turns off the transcription of genes in a concerted manner through differentiation and maturation processes [94]. PI3K/AKT signaling also induces upregulation of the activity of the TET enzymes producing active DNA demethylation. This activation allows differentiation genes, such as the *Ngn1*, to be activated and promotes neuronal stem cell (NSC) commitment to the neural lineage [94].

The regulation of DNA methylation and demethylation processes needs to be tightly controlled through adulthood. Recent studies in the AD model 5xFAD report that DNA methylation is increased when Aβ begins to accumulate and this increase is accompanied by the upregulation of DNMTs [95]. Nevertheless, it is not surprising that different studies report different findings when analyzing post-mortem brains of patients diagnosed with AD. One of the first groups that studied these markers in human brains reported a global decrease in 5mC and 5hmC in the hippocampus, particularly in the CA1 region and the dentate gyrus [96]. However, another study reported hypermethylation of autosomal differentially methylated regions that also contained altered histone methylation markers [97]. More specifically, the promoters of genes associated with plasticity, such as BDNF, have been found to have high levels of methylation in patients with AD, which also correlates with a reduction in the mRNA or protein levels of BDNF [98]. The authors of the respective manuscripts discuss that the differences in their findings might be due to the nature of either the genetic background of the transgenic mice or the stage of AD in patients upon death. They further discuss that, at least in transgenic mice, when they compared aged mice to young mice, they found that the levels of epigenetic markers are upregulated. Nevertheless, the findings demonstrate that the regulation of the methylation/demethylation of DNA is affected by aging and AD. Another possibility to be considered is the particular state of PI3K/AKT activation and the levels of growth factors that are able to activate this signaling pathway.

**Figure 2:**
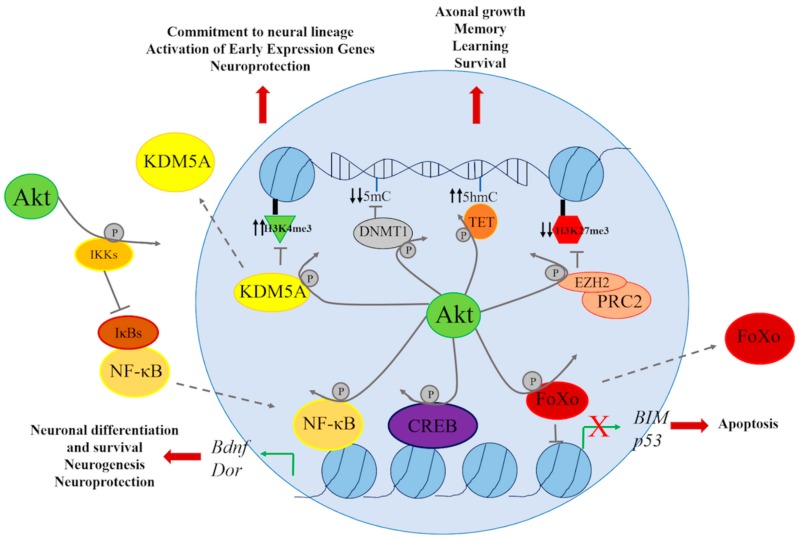
AKT phosphorylates several transcription factors, including NF-κB, CREB, and FOXO, implicated in the transcription of genes regulating neuronal differentiation, neuroprotection, neurogenesis, and apoptosis, such as Bdnf, Dor, BIM, and p53. PI3K stimulation can also inhibit (KDM5A, DNMT1, and EHZ2) or activate (TET) chromatin-associated proteins leading to upregulation or repression of genes implicated in neural differentiation, axonal growth, memory, and learning through altering chromatin marks in histone tails of DNA modifications.

Altogether, this evidence points to the important role of PI3K/AKT signaling in the regulation of gene transcription in the brain throughout life in healthy and diseased states. Although this pathway does not directly impact chromatin, it regulates several transcription factors and chromatin remodeling proteins and complexes that can alter the balance between heterochromatin and euchromatin. Furthermore, whenever this signaling pathway is altered, as in aging or AD, aberrant epigenetic signatures appear that deregulate gene expression, which eventually leads to neurodegeneration (Figure 2).

## 5. Vesicle Recycling and Neurotransmission

**Figure 3:**
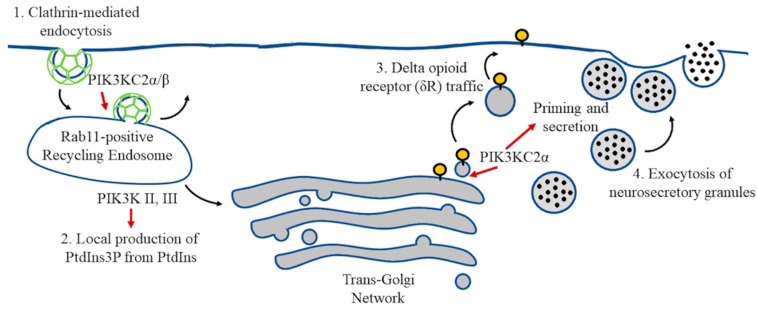
PIK3KC2α and β are associated with clathrin-coated endocytic intermediates (1.) while PIK3K II and III participate in the local production of PtdIns3P at recycling endosomes (2.). PI3KC2α regulates priming and secretion of large dense core vesicles at chromaffin cells (3.) and PI3KC2α is required for Delta opioid receptor (δR) trafficking [4].

In addition to the significant role of class I PI3Ks in the brain and the vast knowledge around this family of PI3K, less is known about the important role of the class II and class III PI3Ks. The action of the two class II PI3Ks (PI3K-C2α, β) has been implicated in clathrin-mediated endocytosis, glucose transport, insulin secretion, neurosecretory granule release, muscle contraction, and cell migration [99]. At present, there is no clear evidence that class II PI3Ks are involved in AKT activation, although PI3K-C2β becomes activated by some growth factors, such as epidermal growth factor (EGF) and platelet-derived growth factor (PDGF), associated with RTKs [100]. Class II and III PI3Ks catalyze the conversion of PtdIns to PtdIns3P, which is a major constituent of endosomal membranes. PtdIns3P is an intracellular second messenger and a hallmark of many signaling pathways controlling proliferation, growth, invasion, and vesicle trafficking [101]. Distinctively, vesicle trafficking encompasses communication between endosomal compartments, which can assist in signaling transduction and act as integrators and processors. Moreover, recycling and retrieval of soluble or membrane-embedded protein cargos from the endosome to the trans-Golgi network or the plasma membrane determine the concentration of important molecules at the cell surface. Therefore, fine regulation of endosomal protein sorting and trafficking must occur to maintain neuronal homeostasis, synapse formation [102], or avoidance of neurodegeneration [103,104]. Although the role of class II PI3Ks in endocytic vesicle recycling is relevant, there are few studies on how the function of class II PI3Ks may impact neurotransmission in the nervous system. PI3K-C2α is present in clathrin-coated vesicles of isolated brains [105] and binds to clathrin-mediated vesicles, which locally produce phosphoinositide in growing clathrin-coated pits [106]. In PC12 cells, activation of PI3K-C2α enhances the exocytosis of neurosecretory granules, whereas transfection of PC12 cells with a catalytically inactive PI3K-C2α inhibits this process [107]. Remarkably, PI3KC2α not only participates in the exocytosis of neurosecretory granules but also in the exocytosis of cargos transported from the trans-Golgi network, such as delta opioid receptor (δR), which requires PI3KC2α for surface trafficking. Inhibition of PI3Ks with wortmannin causes the retention of δR at the Golgi complex, as shown by immunofluorescence colocalization of TGN-38 (a trans-Golgi network marker) and (GFP)-δR [108] (Figure 3). On the other hand, phosphoinositides generated by class II PI3K and class III PI3K in *Drosophila* are necessary for the rapid appearance and long-term maintenance of pre-synaptic homeostatic potentiation through the enhancement of presynaptic vesicle recycling in *Drosophila* [109]. The class II PI3K homologous gene mutant disrupts the generation of PtdIns3P, which determines the early endosomal membrane required for the recruitment of Rab11 to recycling endosomes. Similarly, class III PI3K orthologue Vps34 and postsynaptic Rab11 knockdown also block the expression of trans-synaptic potentiation, demonstrating a new post-synaptic signaling depending on the formation of PtdIns3P by class II and III PI3Ks [109].

## 6. Class III PI3Ks in Neuronal Autophagy

The class III PI3Ks mainly control the trafficking of intracellular vesicles in the context of autophagy, endocytosis, and phagocytosis. The Beclin 1-Vps34 complex is a core component of class III PI3Ks, and binds Atg14L or UVRAG to control different steps of autophagy in mammalian cells. It is not clear which extracellular stimuli are able to activate this complex although some evidence points to the role of nutrients such as glucose and amino acids [110]. The Beclin 1-Vps34 complex is essential for mouse development and viability [111]. Autophagy and endosomal processes are essential for maintaining neuronal stability [112,113]. In a recent study, it was reported that class III PI3K-deficient mice abnormally accumulate protein aggregates, vacuoles, and endosomes in large diameter sensory neurons and induce neurodegeneration [111]. Class III PI3K also mediates the endosome–lysosome degradation to promote axon pruning during development [114]. However, it must be mentioned that autophagy is a complex mechanism that also implicates regulation by PI3K/AKT/mTOR signaling.

## 7. Conclusions

PI3K-mediated signaling is a central node that coordinates a variety of complex events that lead to changes in neuronal development, growth, energy metabolism, and survival. All these functions are carried out through the integration of upstream signals and the downstream control of gene expression. The complexities of function require further studies to better understand the specific regulation of different PI3K enzymes in different metabolic contexts that may have implications in neuronal physiology in healthy and diseased states. These studies will allow for future advances in the understanding of how PI3K signaling is designed and implemented at the cellular and molecular levels to control basic neuronal processes, as well as for the therapeutic development of drugs targeting different PI3K isoforms for treating brain pathologies.
